# Changes in bud morphology, growth-related genes and nutritional status during cheliped regeneration in the Chinese mitten crab, *Eriocheir sinensis*

**DOI:** 10.1371/journal.pone.0209617

**Published:** 2018-12-26

**Authors:** Cong Zhang, Xiao-zhe Song, Qian Zhang, Yang-yang Pang, Jia-huan Lv, Bo-ping Tang, Yong-xu Cheng, Xiao-zhen Yang

**Affiliations:** 1 National Demonstration Center for Experimental Fisheries Science Education; Key Laboratory of Freshwater Aquatic Genetic Resources, Ministry of Agriculture; Engineering Research Center of Aquaculture, Shanghai Ocean University, Shanghai, China; 2 Jiangsu Key Laboratory for Bioresources of Saline Soils, Jiangsu Synthetic Innovation Center for Coastal Bio-agriculture, Jiangsu Provincial Key Laboratory of Coastal Wetland Bioresources and Environmental Protection, School of Ocean and Biological Engineering, Yancheng Teachers University, Yancheng, China; Zhejiang University College of Life Sciences, CHINA

## Abstract

During pond culture of *Eriocheir sinensis*, a high limb-impairment rate restricts the industry development and quality. Therefore, research on limb autotomy and regeneration has important practical significance for the industrial development and basic biology of *E*. *sinensis*. This study evaluated the changes in bud morphology, growth-related gene expression and nutritional status during cheliped regeneration in *E*. *sinensis*. The study found that the new cheliped was pre-formed in the bud and then regenerated with the completion of molting of *E*. *sinensis*. The new cheliped was similar in morphology to the normal cheliped after the first molting but smaller in size. The qRT-PCR results of growth-related genes showed that the expression levels of *EcR*-mRNA (ecdysteroid receptor) and *Chi*-mRNA (chitinase) were significantly up-regulated, whereas the expression of *MIH*-mRNA (molt-inhibiting hormone) was significantly down-regulated (*P < 0*.*05*). The nutritional status during the regeneration process showed that the hepatopancreas total lipid content decreased significantly within 28 days and was significantly lower in the autotomy group than in the control group at 14 d and 21 d (*P < 0*.*05*). The hepatopancreas fatty acid composition results showed that saturated fatty acids (SFA), highly unsaturated fatty acids (HUFA) and n-3/n-6 were significantly higher in the autotomy group than in the control group at 21 d (*P < 0*.*05*), whereas the ∑ n-6 PUFA and ∑ n-3 PUFA at 1 d and 7 d, and the monounsaturated fatty acid (MUFA) at 28 d in the autotomy group were significantly lower than in the control group (*P < 0*.*05*). Moreover, the levels of eicosatetraenoic acid (ARA), eicosapentaenoic acid (EPA) and docosahexaenoic acid (DHA) showed that DHA was significantly lower at 7 d and significantly higher at 21 d in the autotomy group than in the control group (*P < 0*.*05*), whereas ARA and EPA were not significantly different between the two groups. Muscle L-tryptophan content was significantly lower at 1 d and significantly higher at 7 d in the autotomy group than in the control group (*P < 0*.*05*). These results indicate that during the cheliped regeneration process, crabs could accelerate molting and regeneration by regulating growth-related gene expression (e.g., *EcR*-mRNA and *MIH*-mRNA) and nutrient metabolism (e.g., lipid metabolism).

## Introduction

In the face of threats from other animals or environmental stress, many animals take active measures to respond positively in the natural environment. Autotomy, as a congenital, highly effective reflexive response, is very useful in the process of escaping from danger and avoiding threats[1,2]. Although autotomy can let animals escape from danger temporarily, when the autonomous animals are threatened again, it is generally difficult to escape again. In addition, autotomy also brings many negative effects on organisms, such as long-term loss of energy [3], immunity and antibacterial response [4]. Therefore, the best way to compensate for autotomy costs is limb regeneration. In nature, animals with limb autotomy generally achieve limb regeneration after a period of time. However, limb regeneration depends on the gender [5], age [6], molting period [7,8], etc. Study on the autotomy and regeneration of crustaceans have been widely studied, such as feeding rate [9], growth rate [10], and immune function [4,11].

The Chinese mitten crab, *Eriocheir sinensis*, is an important aquaculture species in China. The normal culture cycle of crabs usually takes two years [12]; in the juvenile crab culture process, various factors can lead to limb autotomy, such as predation, fighting behavior, defense and foraging, unsuccessful or unsynchronized molting, high-density farming, or artificial harvesting [7,13–15]. A higher limb autotomy rate seriously affects the economic benefit of *E*. *sinensis*. Among them, the most common type of limb injury or autotomy is the loss of a cheliped among decapods [16,17]. Chelipeds play an important role in agonistic interactions, as well as the defense, capture, manipulation, and subjugation of prey in crustaceans [6]. Therefore, cheliped regeneration plays an important role in the culture of *E*. *sinensis*.

Transcriptomic analysis of *Portunus trituberculatus* in limb regeneration showed that limb regeneration appears to be regulated by multiple signalling pathways, the expression of genes involved in muscle growth, moult and immune-related genes up-regulated [18]. And studies reported that the process of crab cheliped regeneration is primarily dependent on the co-regulation of ecdysteroid and molting-inhibiting hormone (MIH); ecdysteroid promotes cheliped regeneration, whereas MIH inhibits cheliped regeneration [19,20]. And chitinase (Chi) plays an important role in the molting cycle of *E*. *sinensis* [21]. Moreover, studies have shown that melanin induced by phenoloxidase (PO) in crustaceans not only participates in wound repair and immune protection, but also promotes the sclerotization of new exoskeleton after molting [22,23]. However, there are no reports on the expression of growth-related genes in different tissue during cheliped regeneration of *E*. *sinensis*.

Cheliped regeneration of *E*. *sinensis* is not only regulated by related genes but also has an important relationship with nutrition storage *in vivo* [12,20]. For crustaceans, growth and molting depend on the level of nutrient accumulation in the body [24]. The level and composition of lipids in *E*. *sinensis* are closely related to their molting, growth and survival [25]. Lipids are the most important energy reserve and biofilm structural material in the juvenile *E*. *sinensis* hepatopancreas and mainly include saturated fatty acids (SFA), monounsaturated fatty acids (MUFA) polyunsaturated fatty acids (PUFA) and highly unsaturated fatty acids (HUFA) [26]. Among them, HUFA such as eicosatetraenoic acid (ARA), eicosapentaenoic acid (EPA) and docosahexaenoic acid (DHA) are not only the main components of phospholipids in the membrane structure [26] but also play an important role in the development of the central nervous system. Adequate lipid storage is a prerequisite for cheliped regeneration. However, no reports have been published on the changes in nutrient dynamics during cheliped regeneration of *E*. *sinensis*. In addition, tryptophan, as an essential amino acid for crustaceans, participates in protein and lipid metabolism and immune regulation in animals [27–30], and promotes the growth of animals [31]. Muscle moisture is also closely related to crustacean nutrition [32]. Moreover, as a precursor of melatonin [33,34], tryptophan has an important relationship with the expression of genes involved in molting in tissues.

In this study, we observed the morphological and biochemical component changes of cheliped buds during the regeneration process, and evaluated the expression of growth-related genes in tissues, changes of hepatopancreas nutrition status, muscle moisture and L-tryptophan content, with the aim of providing a practical basis for the nutritional support of cheliped regeneration and enriching the knowledge of cheliped regeneration of *E*. *sinensis*.

## Materials and methods

### Experimental crabs

All experimental protocols were reviewed and approved by the Animal Bioethics Committee, Shanghai Ocean University, China. In July 2017, 280 hard-shelled crabs just after molting and limb-intact *E*. *sinensis* (*Crustacea*; *Decapoda*; *Grapsidae*) juvenile crabs (22.45 ± 4.68 g), were obtained from the earth pond at the Chongming research base of Shanghai Ocean University (Shanghai, China), to be used experimentally. Juvenile crabs were acclimated in 60-L ultra-clear glass tanks; each tank was supplied with continuous aerated fresh water at 26°C –28°C, pH 7.84 ± 0.08, DO concentration 6.3 ± 0.4 mg/L, salinity 0.3%, total ammonia 0.36 ± 0.03 mg/L, chloride level 136 ± 15 mg/L, and basal nitrite <0.05 mg/L^-1^ and natural photoperiod conditioning for one week. The crabs were fed once a day with a commercial crab diet.

### Experimental design

A total of 240 healthy, limb-intact crabs were selected and randomly divided into two groups (40 crabs for each group and in triplicate): (1) control group: limb-intact without any treatment; (2) autotomy group: autotomy left cheliped, which was achieved by gently grasping the limbs using the researcher’s fingers, and the crab would spontaneously autotomize the corresponding limbs. Before cheliped autotomy, the crabs were anesthetized with ice. The crabs were returned to the aerated water in monoculture systems immediately and the aquaculture environmental conditions as described above.

### Sample collection

The experiment was completed after 28 days, and the molting and deaths of the two groups were recorded daily and calculated at the end of the experiment. Three individuals were randomly taken from each group at 1 d, 7 d, 14 d, 21 d and 28 d, for sample collection and anesthetized on ice before sampling.

Crab cheliped buds and basal tissues were observed and photographed under a dissecting microscope, and then, they were used to determine chitin and crude protein content. Hemolymph was drawn with a sterile 1-ml syringe from the unsclerotized membrane of the right third pereopod and was diluted 1:1 with sterile anticoagulation agent (trisodium citrate 30 mM, NaCl 338 mM, glucose 115 mM, EDTA 10 mM), and then, the mixture was centrifuged at 3500 r/min for 10 min to collect the hemocyte and cell-free hemolymph and stored at -20°C to determine the activity of PO.

A total of 500 μL hemolymph was drawn again (procedure as described above) and diluted 1:1 with sterile anticoagulation agent immediately and centrifuged at 12 000 r/min for 10 min to collect the hemocyte, hepatopancreas, epidermal and pereopod muscle samples, which were stored at -80°C for RNA isolation. The rest of the hepatopancreas samples and abdominal muscles were stored at -20°C for evaluation of nutrition related parameters.

### Chitin, crude protein and PO activity determination

#### Crude protein and chitin

The determination of chitin and crude protein was slightly modified according to Tian *et al* (2013) [35]: crab cheliped buds and basal tissues (as described in “2.3 Sample collection”) were accurately weighed using an ultramicrobalance (W_0_) and then boiled in a 10% NaOH solution for 1 hour to remove protein. Then, 95% ethanol, 50% ethanol, and distilled water were each used to wash the samples 3 times, in that order, and the weight was determined after samples were dried at 60°C (W_1_). The tissue was then soaked with 3.6% HCl for 15 min to obtain the transparent chitin, which was dried at 60°C and weighed (W_2_).

Crude protein relative content = (W_0_ –W_1_) / W_0_ × 100%

Chitin relative content = W_2_ / W_0_ × 100%

#### PO activity

The PO activity in hemocyte lysate (HL) and cell-free hemolymph (CFH) were measured using a commercial kit (Nanjing Jiancheng Bioengineering Institute, Nanjing, China) in accordance with the manufacturer’s protocols.

### Expression of the *EcR*, *MIH* and *Chi* gene level: Quantitative RT-PCR

Total RNA was extracted from the hemocyte, hepatopancreas, epidermal and pereopod muscle tissues using RNAiso plus reagent (RNA Extraction Kit, TaKaRa, Japan) according to the manufacturer’s protocol. The concentration and quality of the total RNA were estimated by micro-volume ultraviolet-visible spectrophotometer (Quawell Q5000; Thmorgan, China) and agarose-gel electrophoresis, respectively, and reverse transcribed with the PrimeScript RT reagent Kit (Perfect Real Time, TaKaRa, Japan) according to the manufacturer’s protocol. The cDNA obtained was diluted to 1:2 with double-distilled water and used as qRT-PCR template. Relative quantification was performed using the ABI 7500 Real-Time PCR System (Life Technology, USA) with ChamQ Universal SYBR qPCR Master Mix (Vazyme Biotech Co.,Ltd, Nanjing, China) kits using the following program: 95°C for 30 s; 40 cycles at 95°C for 5 s, 60°Cfor 34 s; followed by a melting curve at 95°C for 15 s, 60°C for 1 min, 95°C for 15 s. The PCR primer sequences for *EcR*, *MIH and Chi* are shown in Table 1 (Sangon Biotech Co., Ltd. Shanghai, China). β-actin was used as the internal control and assays were performed in triplicate for every sample. Relative changes in gene expression levels were determined by 2^-ΔΔCt^ method.

**Table 1:** Primer information for quantitative real-time polymerase chain reaction.

Primers	Sequences (5’-3’)
***EcR*-F**	GGGCATCGGGCTACCACTACAAC
***EcR*-R**	GGCACTGAGACTCGGGCACAACA
***MIH*-F**	TGAAGACTGCGCCAACATCT
***MIH*-R**	GCTCGTCAGGGTAGGTGGTG
***Chi*-F**	GAGCCCTACGTCTACAGCATCAC
***Chi*-R**	GGTCTCAACACTCCAAACCATCA
***β-actin* -F**	TCATCACCATCGGCAATGA
***β-actin* -R**	TTGTAAGTGGTCTCGTGGATG

### Determination of nutritional related parameters

#### Hepatopancreas total lipid and fatty acid composition determination

The extraction of hepatopancreas total lipid was carried out with a chloroform and methanol mixture (2:1, v/v) modified according to Folch *et al*. (1957) [36]. Fatty acid analysis was performed according to the method of Wu et al. [37], using 14% boron trifluoride-methanol (v/v) for methyl esterification of total lipids [38]. The instrument used was an Agilent 6890 gas chromatograph, and the capillary column was fitted with an HP-5.5% Phenyl Methyl Siloam (30.0 m × 0.25 mm, Agilent 19091J-413, USA). The injector temperature was 250°C, and the detector temperature was 280°C. The column temperature was initially held at 60°C, followed by an increase at a rate of 50°C/min to 170°C, then to 180°C at 2°C/min for 2 min, then to 230°C at 3°C/min for 1 min, and then to 240°C at 1°C/min for 1 min., total time was approximately 46.2 min for all fatty acids peak. The carrier gas was helium with a flow velocity of 25 mL/min. Peaks were identified by comparing retention times with known standards (Sigma Chemical Co, St. Louis. MO, USA), and individual fatty acids were quantified by reference to the internal standard (C19:0). Fatty acid composition was expressed as a percentage for each fatty acid of the total fatty acid [39].

#### Muscle moisture and L-tryptophan content determination

To prevent high temperatures from destroying amino acids in the muscle tissue, we used a vacuum freeze-drying method to measure the abdominal muscle moisture. Details are as follows: a 5-ml Eppendorf tube was dried in a 55° C air dry oven, removed and then cooled in a dry environment. The weight of the Eppendorf tube + wet muscle was accurately determined with an electronic balance (W_3_), then transferred to a -40°C freezer for 2 hours, and then placed in a vacuum freeze drier (-40°C) for 48 hours until completely dried and accurately weighed (W_4_).

muscle moisture = W_3_ –W_4_

The freeze-dried muscles described above were used for the determination of L-tryptophan content. Determination of L-tryptophan content is based on the National Standard of the People's Republic of China, "determination of amino acids in feed" (GB/T 18246–2000), using alkaline hydrolysis pretreatment, and the of L-tryptophan content in muscle was determined by reversed-phase high-performance liquid chromatography (RP-HPLC). A C18 (μ- Bondapak Cl8 column, diameter 25 cm × 4.6 mm) column was selected, the mobile phase was composed of sodium acetate buffer + methanol = 95+5, the flow rate was 1.5 mL/min, ultraviolet (UV) detection wavelength was 280 nm, the injection volume was 15 μL, and the column was at room temperature.

### Statistical analyses

Data are presented as the mean values ± standard deviation (SD). The percentage values (dependent variable) were arcsine transformed before analysis. The effects of treatment were statistically analyzed using an analysis of variance (one-way ANOVA, LSD and Duncan analysis), and a *P*-value < 0.05 was considered significant. All statistical analyses were performed using SPSS 20.0 software (Chicago, USA; Version 20.0).

## Results

### Cheliped bud morphology and composition analysis

#### Cheliped bud morphology

**Fig 1:**
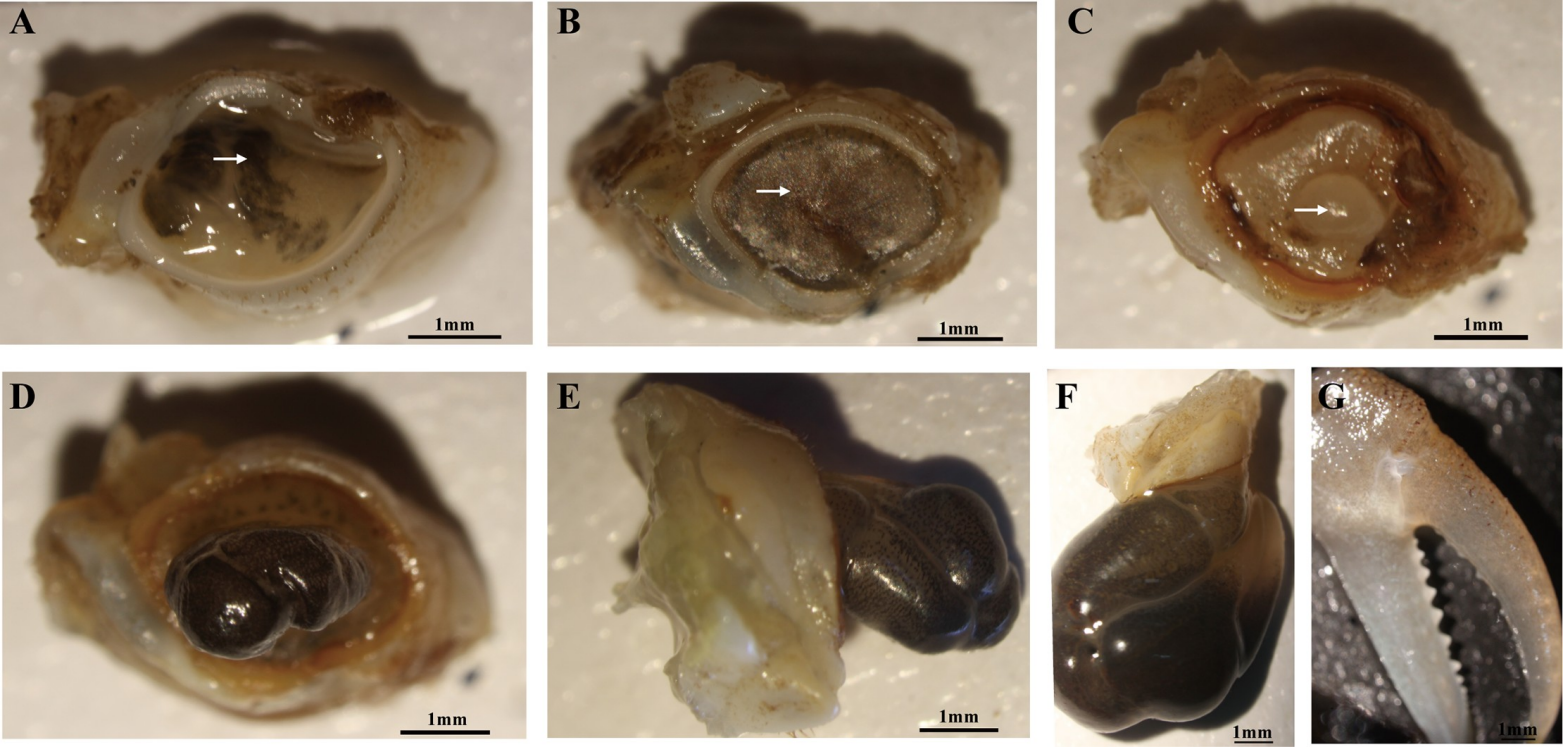
Morphological changes of *E*. *sinensis* cheliped bud regeneration within 28 days observed with a dissecting microscope. (A): photographed immediately after autotomy, top view; black material represented by arrows. (B): 1 day after autotomy, top view; dark brown biofilm represented by arrows. (C): 7 days after autotomy, top view; transparent hemisphere-like crystalline encrustations represented by arrows. (D): 14 days after autotomy, top view; (E): 14 days after autotomy, side view; (F): 21 days after autotomy, side view; (G): 28 days after autotomy.

Fig 1 shows the morphological changes of the cheliped regeneration process of *E*. *sinensis* after autotomy. When the cheliped was autotomized, in order to prevent the continuous loss of hemolymph, black material (represented by arrows) rapidly accumulated at the wound site (Fig 1A) and a layer of dark brown biofilm (represented by arrows) was formed to cover the wound surface (Fig 1B). A few days later, transparent hemisphere-like crystalline encrustations (represented by arrows) broke out of the dark brown biofilm at the autotomy site (Fig 1C), followed by rod-like growth and prolongation, and the surface was fully covered with black material (Fig 1D, 1E and 1F). However, the surface of the bud was soft throughout the entire process and no hardened shell was formed. The new cheliped was pre-formed in the bud and then regenerated with the completion of molting (Fig 1G). The morphology and function of the regenerated new cheliped was similar to the original cheliped, but smaller in size.

#### Chitin and crude protein content

**Fig 2:**
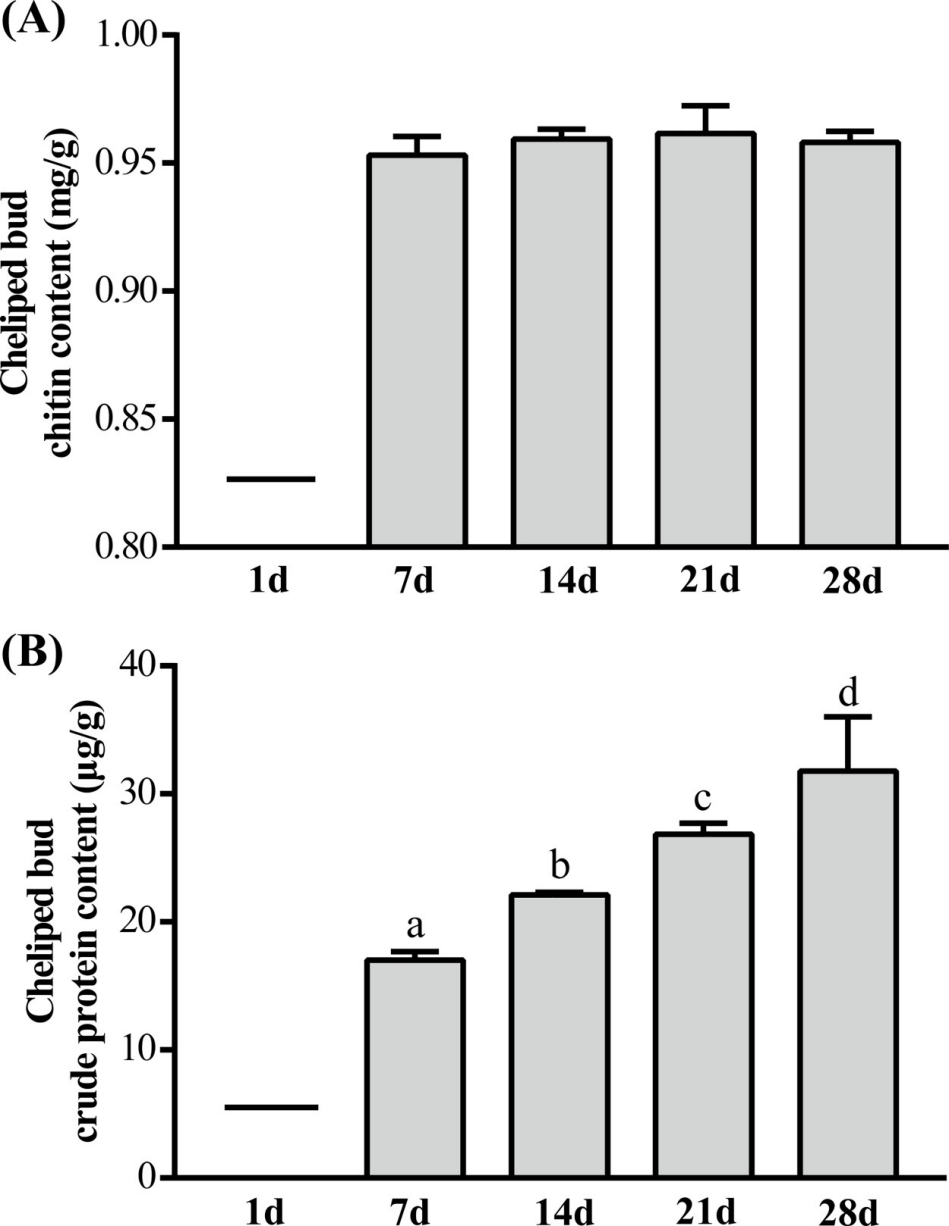
Changes of chitin and crude protein content in regenerated cheliped bud of *E*. *sinensis* within 28 days after treatment. (A) cheliped bud chitin content; (B) cheliped bud crude protein content. The values are expressed as the means ± SD (n = 9). Different letters above the columns represent the significant differences with the same treatment at different times (*P<0*.*05*).

Since no buds were grown on the first day after autotomy, there was no information on the determination of chitin and crude protein at 1 d (Fig 2A and 2B). There were no significant changes of chitin content in the buds during regeneration of the cheliped (Fig 2A). However, the content of crude protein in buds increased significantly within 28 days (*P < 0*.*05*) (Fig 2B).

#### PO activity

**Fig 3:**
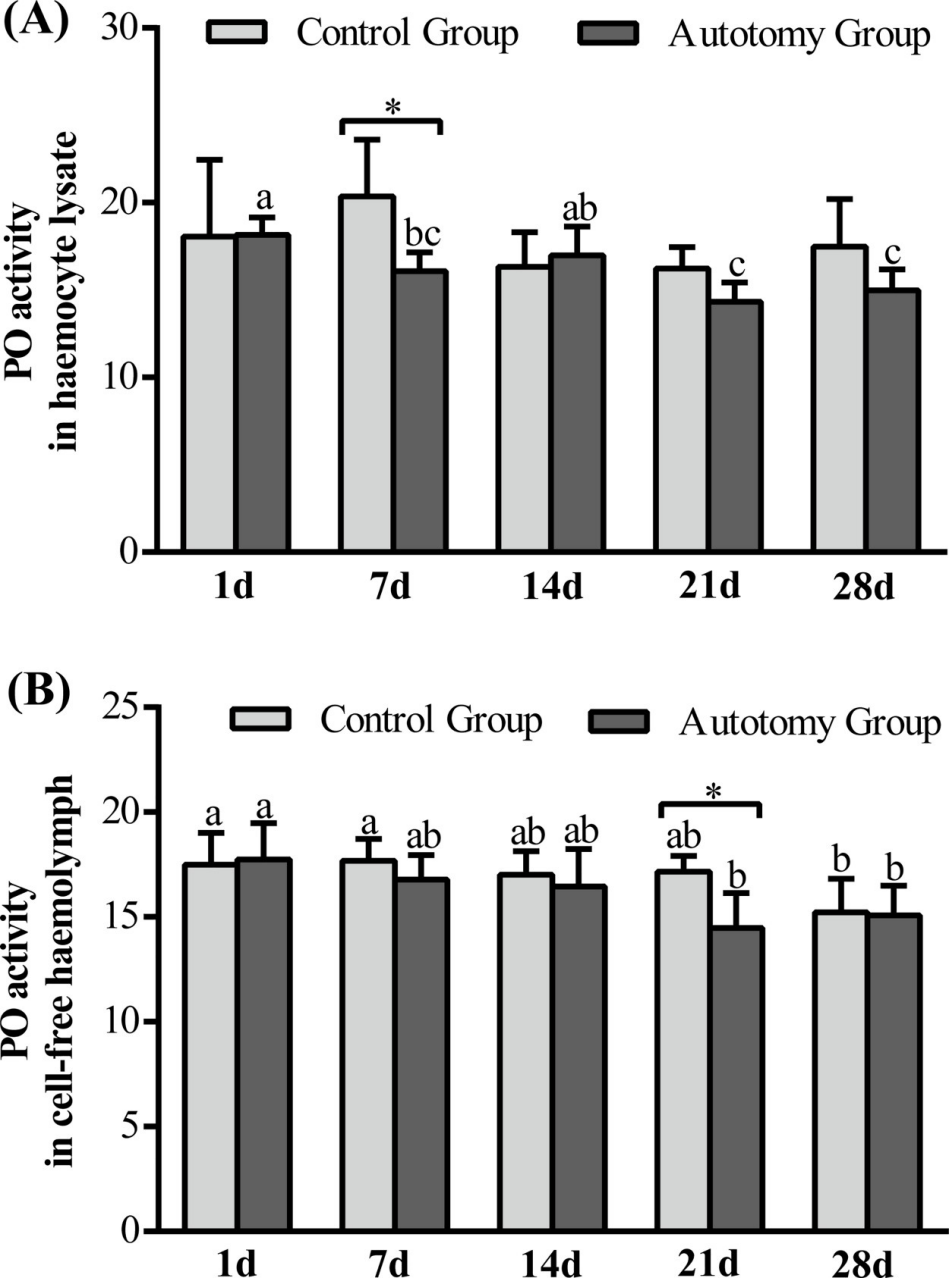
Changes of PO activity in regenerated cheliped bud of *E*. *sinensis* within 28 days after treatment. The values are expressed as the means ± SD (n = 4). (A): PO activity in HL; (B): PO activity in CFH. Different letters above the columns represent significant differences with the same treatment at different times (*P<0*.*05*). * represents significant differences between control group and autotomy group at the same time point (*P < 0*.*05*).

In HL, PO activity was not significantly changed in the control group, whereas it decreased significantly at 7 d, 21 d and 28 d compared with 1 d after autotomy (*P < 0*.05) (Fig 3A). Moreover, the t-test results showed that PO activity in the autotomy group was significantly lower than that in the control group at 7 d (*P<0*.*05*) (Fig 3A). However, in CFH, PO activity was decreased significantly at 28 d compared with other days in the control group (*P < 0*.*05*), whereas it was decreased significantly at 21 d and 28 d compared with other days in the autotomy group (*P < 0*.*05*) (Fig 3B). Moreover, the t-test results showed that the PO activity in the autotomy group was significantly lower than that in the control group at 21 d (*P < 0*.*05*) (Fig 3B).

### Expression levels of growth-related genes: *Chi*, *EcR* and *MIH*

#### Expression of the *Chi* gene levels

**Fig 4:**
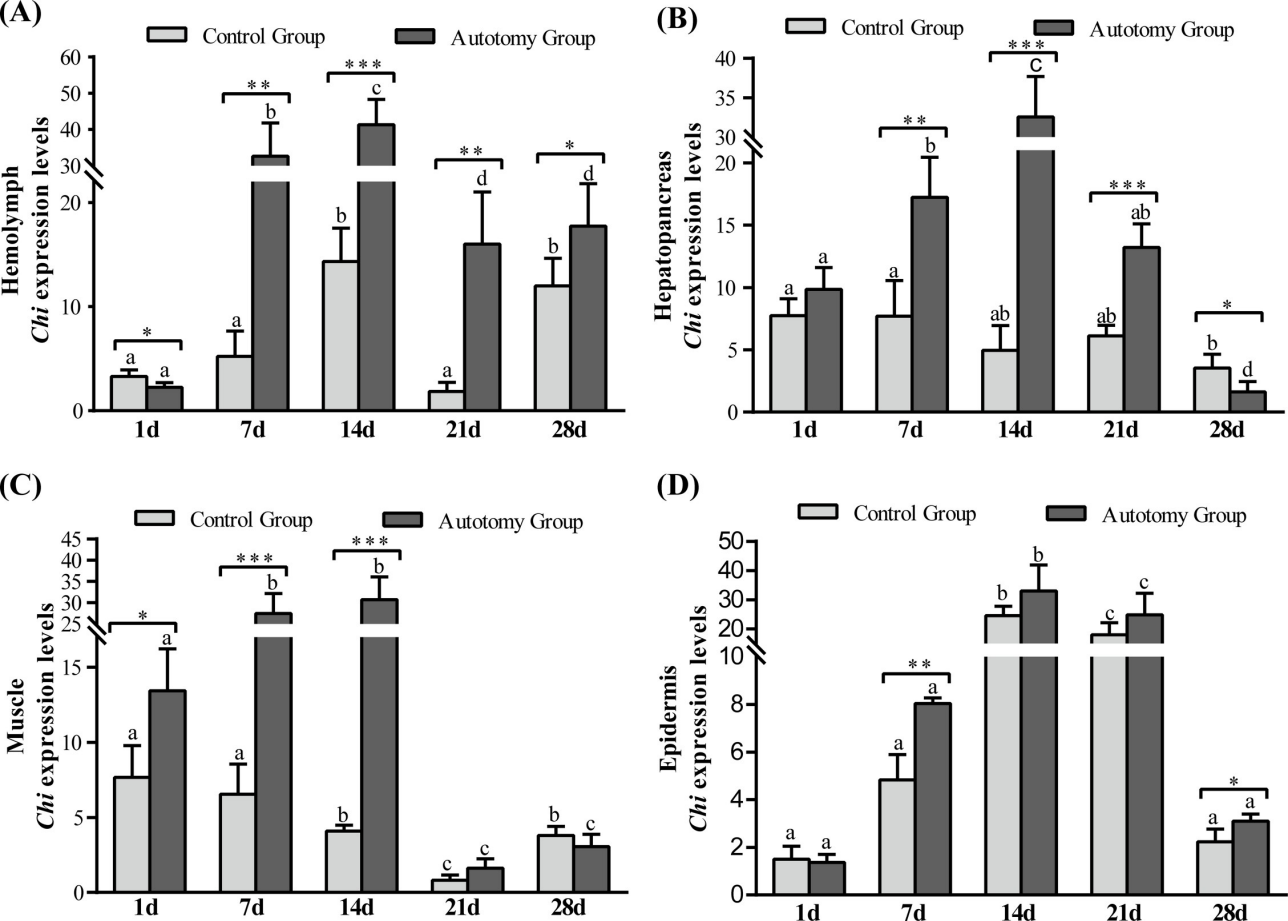
Expression level of *Chi* gene normalized to *β*-actin in hemolymph, hepatopancreas, muscle and epidermis of *E*. *sinensis* with different treatments. (A) hemolymph; (B) hepatopancreas; (C) pereopod muscle; (D) epidermis. The values are expressed as the means ± SD (n = 4). Different letters above the columns represent significant differences with the same treatment at different times (*P<0*.*05*). * represents significant differences between control group and autotomy group at the same time point (* *P < 0*.*05*, ** *P<0*.*01*, *** *P<0*.*001*).

The expression levels of *Chi* gene in different tissues showed a tendency to up-regulate first, then down-regulate both in the control group and the autotomy group (Fig 4). Compared with the control group, the expression levels of *Chi* gene were significantly higher in the autotomy group at 7 d (*P<0*.*01*), 14 d (*P < 0*.*001*), 21 d (*P < 0*.*01*) and 28 d (*P<0*.*05*) in hemolymph (Fig 4A). The expression levels of *Chi* gene were significantly up-regulated in the autotomy group at 7 d (*P < 0*.*01*), 14 d (*P < 0*.*001*) and 21 d (*P < 0*.*001*) compared with the control group, whereas it was significantly lower at 28 d (*P<0*.*05*) in the hepatopancreas (Fig 4B). Compared with the control group, the expression levels of *Chi* gene were significantly higher in the autotomy group at 1 d (*P<0*.*05*), 7 d (*P < 0*.*001*) and 14 d (*P < 0*.*001*) in pereopod muscle (Fig 4C). Moreover, the expression levels of *Chi* gene were significantly higher than that in the control group at 7 d (*P<0*.*01*) and 28 d (*P<0*.*05*) in epidermis (Fig 4D).

#### Expression of *EcR* gene levels

**Fig 5:**
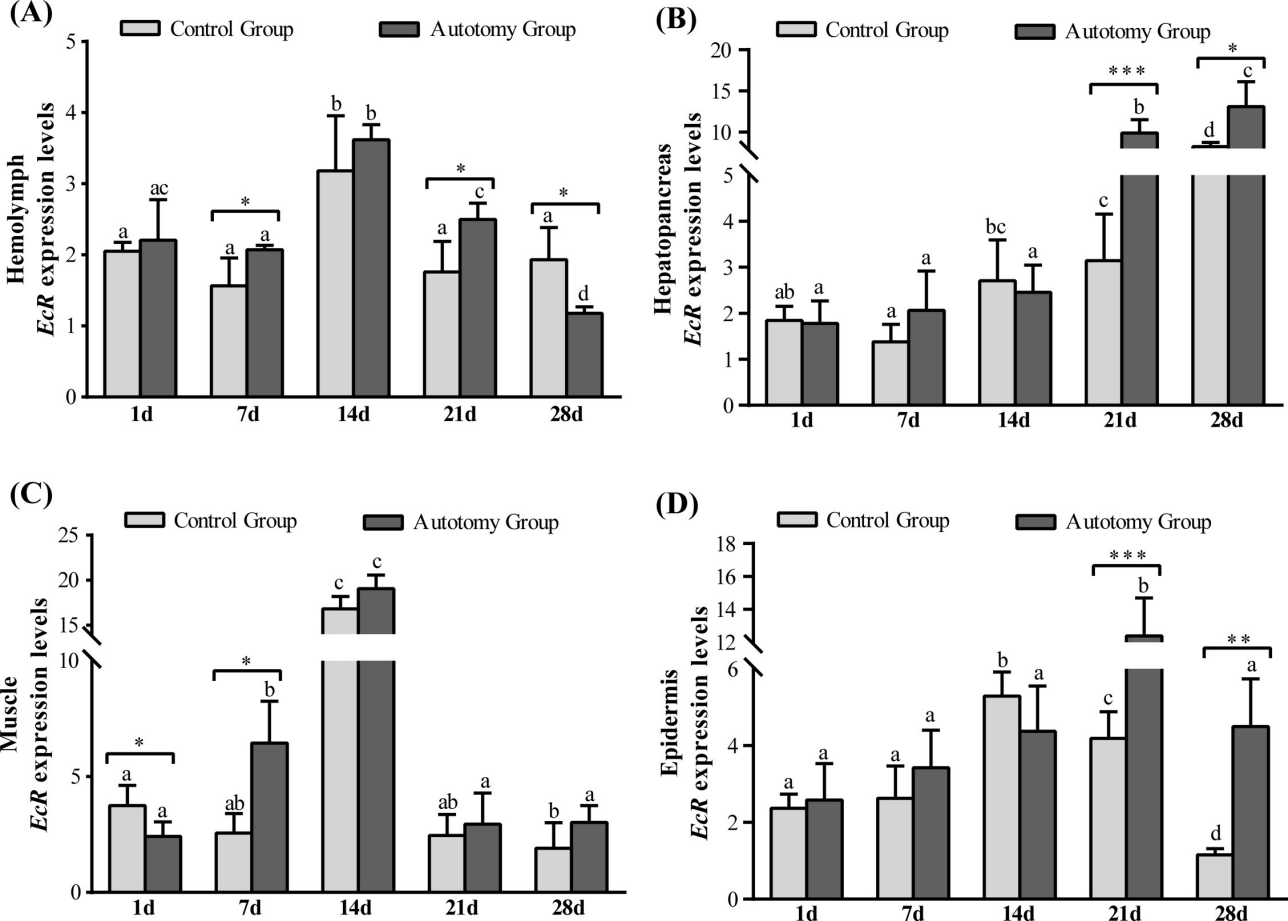
Expression of *EcR* gene normalized to *β*-actin in hemolymph, hepatopancreas, muscle and epidermis of *E*. *sinensis* with different treatments. (A) hemolymph; (B) hepatopancreas; (C) muscle; (D) epidermis. The values are expressed as means ± SD (n = 4). Different letters above the columns represent significant differences with the same treatment at different times (*P<0*.*05*). * represents significant differences between the control group and the autotomy group at the same time point (* *P < 0*.*05*, ** *P<0*.*01*, *** *P<0*.*001*).

Compared with the control group, the expression levels of *EcR* gene were significantly higher in the autotomy group at 7 d (*P<0*.*05*) and 21 d (*P < 0*.*05*), whereas it was significantly lower at 28 d (*P<0*.*05*) in hemolymph (Fig 5A). The expression levels of *EcR* gene were significantly up-regulated in the autotomy group at 21 d (*P < 0*.*001*) and 28 d (*P < 0*.*05*) compared with the control group in hepatopancreas (Fig 5B). Compared with the control group, the expression levels of *EcR* gene were significantly lower at 1 d (*P < 0*.*05*) in the autotomy group, whereas it was significantly higher at 7 d (*P < 0*.*05*) in pereopod muscle (Fig 5C). Moreover, the expression levels of *EcR* gene were significantly higher than that in control group at 21 d (*P<0*.*001*) and 28 d (*P<0*.*01*) in epidermis (Fig 5D).

#### Expression of the *MIH* gene levels

**Fig 6:**
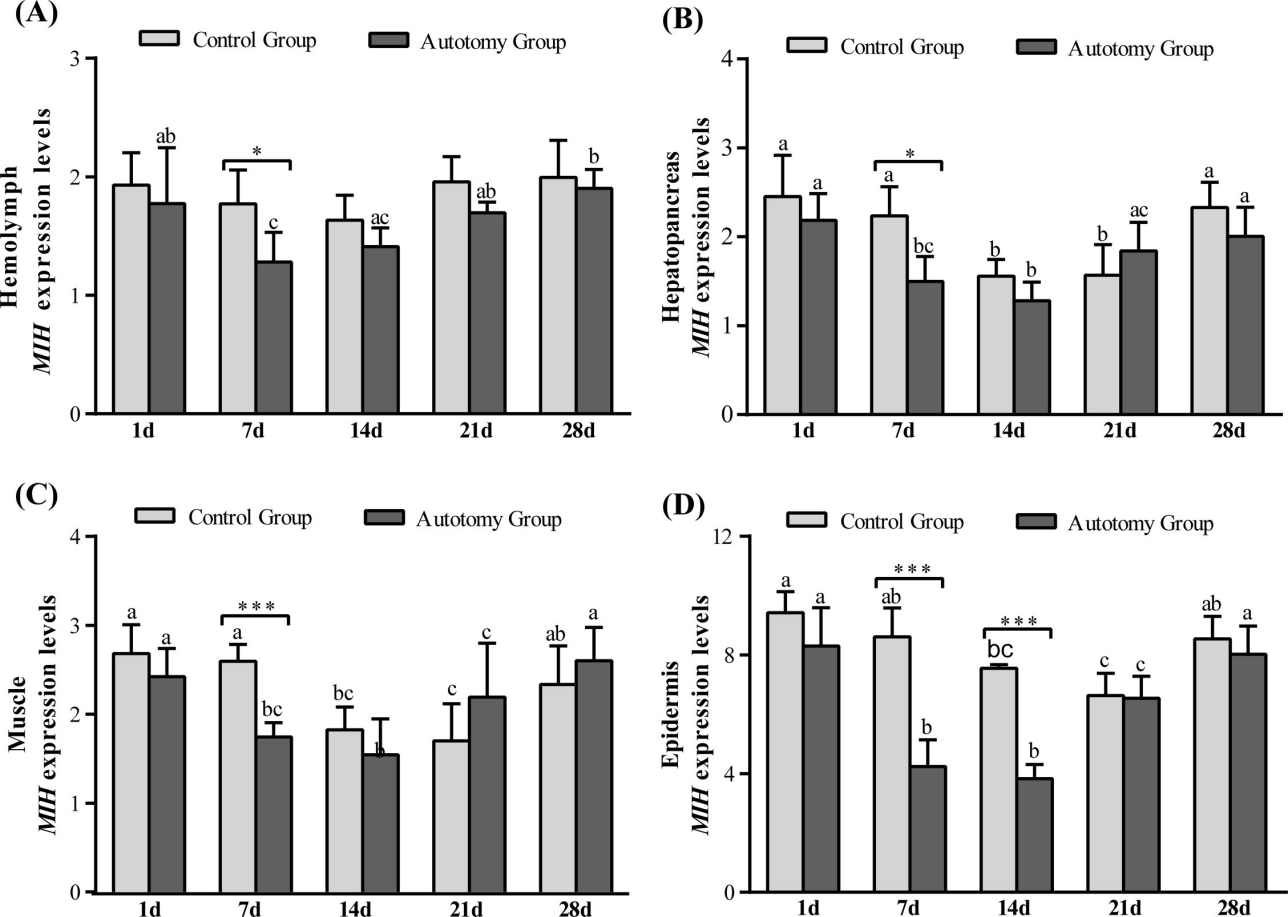
Expression level of *MIH* gene normalized to *β*-actin in the hemolymph, hepatopancreas, muscle and epidermis of *E*. *sinensis* with different treatments. (A) hemolymph; (B) hepatopancreas; (C) muscle; (D) epidermis. The values are expressed as the means ± SD (n = 4). Different letters above the columns represent significant differences with the same treatment at different times (*P<0*.*05*). * represents significant differences between the control group and the autotomy group at the same time point (* *P < 0*.*05*, ** *P<0*.*01*, *** *P<0*.*001*).

The expression levels of *MIH* gene in different tissues showed a tendency to down-regulate first and then up-regulate both in the control group and the autotomy group (Fig 6). Compared with the control group, the expression levels of *MIH* gene were significantly lower in the autotomy group at 7 d in hemolymph (*P<0*.*05*), hepatopancreas (*P<0*.*05*) and pereopod muscle (*P<0*.*001*) (Fig 6A, 6B and 6C). Moreover, the expression levels of *MIH* gene were significantly lower than that in control group at 7 d (*P<0*.*001*) and 14 d (*P<0*.*001*) in epidermis (Fig 6D).

### Nutritional related parameters

#### Hepatopancreas total lipid

**Fig 7:**
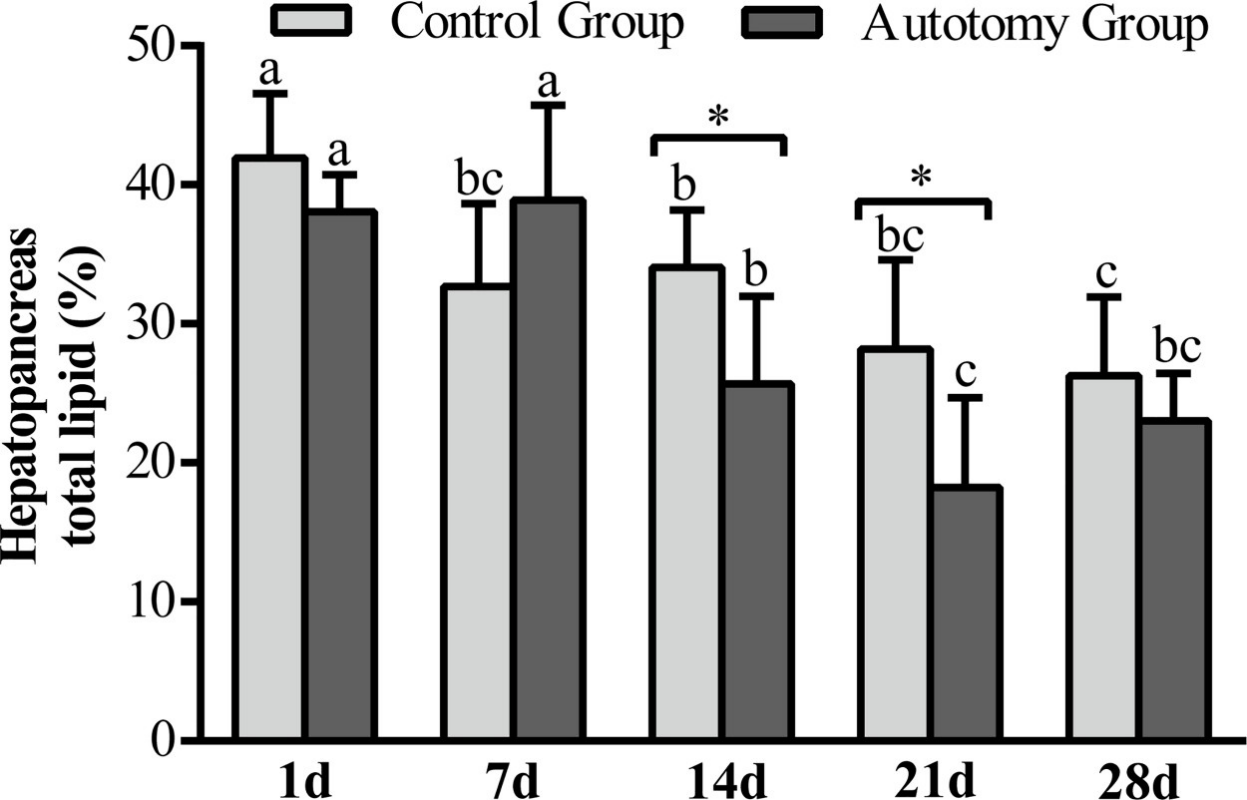
Changes of hepatopancreas total lipid of *E*. *sinensis* within 28 days after treatment. The values are expressed as the means ± SD (n = 5). Different letters above the columns represent significant differences with the same treatment at different times (*P<0*.*05*). * represents significant differences between the control group and the autotomy group at the same time point (* *P < 0*.*05*).

In both the autotomy group and the control group, the hepatopancreas total lipid content showed a tendency to decrease within 28 d (Fig 7). Hepatopancreas total lipid content reached its lowest value at 21 d in autotomy (*P <0*.*05*); the *t*-test results showed that it was significantly lower at 14 d and 21 d in the autotomy group than in control group (*P < 0*.*01*).

#### Hepatopancreas fatty acid composition

The fatty acid composition analysis showed that in the autotomy group, the most abundant saturated fatty acids (SFA) (% total fatty acids) in the hepatopancreas were C16:0 and C18:0; the most abundant monounsaturated fatty acids (MUFA) (% total fatty acids) were C18:1n-7 and C18:1n-9; and the most abundant polyunsaturated fatty acids (PUFA) were C18: 2n-6 (LA) and C22: 6n-3 (DHA), which were similar to the control group (Table 2).

**Table 2:** Fatty acid composition (% total fatty acids) of hepatopancreas of normal and left cheliped autotomy *E*. *sinensis* within 28 days after treatment.

Fatty acid	Control Group					Autotomy Group				
	1 d	7 d	1 4d	21 d	28 d	1 d	7 d	14 d	21 d	28 d
**C14:0**	0.34 ±0.06 ^a^	0.36±0.04 ^a^	0.28±0.09 ^ab^	0.37±0.04 ^a^	0.25±0.11 ^b^	0.50±0.03 ^a^	0.39±0.09 ^b^	0.37±0.10 ^b^	0.35±0.06 ^b^	0.52±0.08 ^a^
**C15:0**	0.13±0.03 ^ab^	0.12±0.04 ^a^	0.13±0.03 ^ab^	0.13±0.03 ^ab^	0.16±0.03 ^b^	0.77±0.11 ^a^	0.15±0.02 ^b^	0.24±0.05 ^a^	0.22±0.05 ^a^	0.13±0.02 ^b^
**C16:0**	7.32±1.41 ^a^	8.18±1.64 ^a^	5.45±0.52 ^b^	5.60±0.97 ^b^	5.10±0.29 ^b^	6.60±0.63 ^a^	6.67±1.22 ^a^	6.07±0.91 ^ab^	5.73±0.57 ^ab^	4.88±1.21 ^b^
**C17:0**	0.13±0.06	0.10±0.00	0.13±0.09	0.11±0.03	0.10±0.03	0.11±0.02 ^ab^	0.12±0.02 ^ab^	0.15±0.06 ^a^	0.10±0.00 ^b^	0.10±0.01 ^b^
**C18:0**	15.40±0.96 ^a^	16.26±1.06 ^ab^	17.21±2.51 ^ab^	17.86±1.15 ^b^	23.97±2.82 ^c^	15.84±1.21 ^a^	16.84±1.45 ^a^	16.31±2.80 ^a^	20.60±1.96 ^b^	20.07±0.50 ^b^
**C20:0**	1.69±0.59	1.44±0.19	1.72±0.35	1.56±0.44	1.55±0.28	1.24±0.37 ^a^	1.10±0.36 ^a^	1.70±0.23 ^b^	1.31±0.11 ^ab^	1.47±0.38 ^ab^
**C22:0**	0.31±0.09 ^b^	0.22±0.04 ^a^	0.18±0.04 ^a^	0.22±0.05 ^a^	0.18±0.04 ^a^	0.42±0.07 ^a^	0.19±0.05 ^b^	0.45±0.05 ^a^	0.24±0.08 ^b^	0.18±0.05 ^b^
**∑ SFA**	25.33±1.62^a^	26.67±2.50 ^a^	25.12±2.03 ^a^	25.84±1.56 ^a^ *	28.93±2.90^b^	25.49±1.81 ^a^	25.47±1.27 ^a^	25.29±2.63 ^a^	28.35±1.95 ^b^ *	27.34±1.66 ^b^
**C16:1**	0.22±0.07 ^a^	0.24±0.09 ^a^	0.20±0.05 ^ab^	0.17±0.03 ^ab^	0.14±0.04 ^b^	0.19±0.06 ^a^	0.18±0.04 ^ab^	0.18±0.02 ^ab^	0.29±0.09 ^c^	0.13±0.03 ^b^
**C17:1n-7**	0.58±0.09 ^a^	1.47±0.18^b^	1.48±0.48^b^	0.22±0.06 ^c^	0.13±0.02 ^c^	0.17±0.03	0.21±0.12	0.20±0.04	0.19±0.01	0.13±0.03
**C18:1n-9**	17.02±4.59 ^a^	18.20±4.82 ^a^	23.45±5.60 ^b^	18.20±3.97 ^a^	13.72±1.50 ^a^	17.46±3.79 ^a^	17.47±4.96 ^a^	17.61±2.99 ^a^	18.08±1.74 ^a^	11.48±1.51 ^b^
**C18:1n-7**	5.29±0.68 ^ab^	6.04±0.97 ^a^	2.96±0.22 ^c^	5.65±1.30 ^ab^	4.58±1.37 ^b^	6.29±1.16 ^a^	3.05±0.67 ^b^	5.51±0.52 ^a^	5.55±0.89 ^a^	3.49±0.53 ^b^
**C20:1n-9**	0.18±0.04 ^a^	0.22±0.05 ^a^	0.47±0.08 ^b^	0.28±0.07 ^c^	0.16±0.03 ^a^	0.28±0.08 ^a^	0.42±0.09 ^b^	0.54±0.02 ^c^	0.58±0.03 ^c^	0.62±0.12 ^c^
**C22:1n-9**	0.29±0.06 ^a^	0.27±0.02 ^a^	0.16±0.04^b^	0.16±0.05^b^	0.21±0.03^b^	0.11±0.02 ^a^	0.16±0.02 ^b^	0.14±0.03 ^b^	0.14±0.00 ^b^	0.13±0.04 ^ab^
**∑ MUFA**	23.57±4.35 ^ab^	26.44±5.10 ^bc^	28.73±6.03^c^	24.68±3.08 ^bc^	18.95±1.80 ^a^ **	24.50±3.03 ^a^	21.48±5.46 ^a^	24.18±2.75 ^a^	24.83±2.60 ^a^	15.98±1.19 ^b^ **
**C18:2n-6 (LA)**	9.99±0.95 ^a^	7.25±0.73 ^b^	9.45±1.98 ^a^	7.25±0.82 ^b^	5.84±1.53 ^b^	6.93±0.86 ^ab^	8.24±1.61 ^a^	7.90±1.45 ^ab^	6.03±1.65 ^b^	5.90±1.55 ^b^
**C18:3n-3 (LNA)**	0.68±0.07 ^a^	0.14±0.03 ^b^	0.27±0.10^c^	0.16±0.02 ^b^	0.15±0.02 ^b^	0.61±0.09 ^a^	0.15±0.03 ^b^	0.12±0.03 ^b^	0.15±0.01 ^b^	0.12±0.03 ^b^
**C20:2n-6**	0.14±0.01	0.13±0.01	0.13±0.01	0.12±0.02	0.15±0.02	0.14±0.04	0.14±0.03	0.16±0.03	0.12±0.01	0.15±0.03
**C20:3n-6**	1.39±0.22	1.67±0.52	1.68±0.39	1.24±0.24	1.26±0.14	1.51±0.27	1.57±0.42	1.49±0.27	1.36±0.31	1.50±0.27
**C20:4n-6 (ARA)**	2.82±0.62 ^ab^	2.70±0.51 ^ab^	3.21±0.69 ^a^	2.19±0.50 ^b^	2.65±0.58 ^ab^	3.10±0.60	3.46±0.91	3.49±0.68	2.69±0.45	3.07±0.98
**C20:3n-3**	0.40±0.09 ^a^	0.38±0.08 ^ab^	0.35±0.08 ^ab^	0.27±0.06 ^b^	0.31±0.09 ^ab^	0.35±0.06	0.34±0.07	0.39±0.10	0.30±0.06	0.34±0.05
**C20:5n-3 (EPA)**	1.56±0.30 ^a^	2.23±0.60 ^ab^	2.00±0.33 ^ab^	2.42±0.96 ^b^	1.92±0.43 ^ab^	1.88±0.41	2.04±0.60	2.01±0.51	2.11±0.26	2.15±0.54
**C22:5n-3**	0.12±0.04	0.15±0.02	0.13±0.05	0.14±0.05	0.11±0.04	0.11±0.02	0.13±0.03	0.12±0.03	0.12±0.02	0.11±0.03
**C22:6n-3 (DHA)**	12.80±0.65 ^a^	12.68±1.39 ^a^ *	12.63±3.13 ^a^	8.75±1.50 ^b^**	11.68±1.46 ^a^	11.45±1.46	10.71±0.75*	10.67±1.12	12.08±1.06 **	11.02±1.96
**∑ n-6 PUFA**	14.34±1.61^a^ *	11.75±1.35^b^	14.46±1.62 ^a^	10.80±1.47^b^	9.90±1.97^b^	11.69±1.53^ab^*	13.40±2.71^a^	13.03±0.90^a^	10.21±1.71^b^	10.62±2.68 ^ab^
**∑ n-3 PUFA**	15.56±0.69 ^a^	15.58±0.94 ^a^ **	15.39±3.24 ^a^	11.74±0.94 ^b^ **	14.17±1.39 ^a^	14.41±1.11	13.38±1.07**	13.31±1.27	14.75±1.00**	13.74±1.43
**∑ HUFA**	19.78±1.20^ab^	19.96±0.87^ab^	20.28±2.80^b^	15.16±1.41^c^**	18.09±1.95^a^	19.03±0.82	18.40±1.79	18.28±0.79	18.81±1.69**	18.31±0.45
**∑ n-3 HUFA**	15.56±0.69 ^a^	15.58±0.94 ^a^ **	15.39±3.24 ^a^	11.74±0.94 ^b^ **	14.17±1.39 ^a^	14.41±1.11	13.38±1.07	13.31±1.27**	14.75±1.00**	13.74±1.43
**∑ n-6 HUFA**	4.22±0.77^c^	4.38±0.94 ^ab^	4.89±1.06 ^a^	3.43±0.72^b^	3.91±0.58 ^ab^	4.62±0.69	5.02±1.32	4.97±0.70	4.06±0.70	4.57±1.22
**n-3 / n-6**	1.09±0.13^a^	1.34±0.20 ^b^	1.06±0.14 ^a^	1.10±0.11 ^a^ *	1.45±0.16 ^b^	1.26±0.25 ^ab^	1.04±0.27 ^a^	1.17±0.08 ^ab^	1.47±0.26 ^b^ *	1.39±0.46 ^ab^
**DHA / EPA**	8.52±2.08^a^	6.03±1.82 ^b^ *	6.39±1.76 ^ab^	4.23±2.12 ^b^	6.39±2.08 ^ab^	6.44±2.05	5.57±1.50*	5.60±1.61	5.83±1.13	5.56±2.16
**ARA / EPA**	1.80±0.11^a^	1.24±0.22^bc^	1.61±0.26 ^ab^	1.02±0.46^c^	1.45±0.53 ^abc^	1.68±0.32 ^ab^	1.78±0.59 ^ab^	1.87±0.76 ^b^	1.31±0.38 ^a^	1.42±0.18 ^ab^

Note: Different letters represent significant differences with the same treatment at different times (*P < 0*.*05*). Significant differences between control group and autotomy group at the same time point

* *P < 0.05*

** *P < 0.01*.

SFA was significantly increased at 28 d (28.93±2.90%) compared with 1 d (25.33±1.62%) (*P < 0*.*05*) in the control group, and it was significantly higher at 21 d (28.35±1.95%) and 28 d (27.34±1.66%) than 1 d (25.49±1.81%) in the autotomy group (*P < 0*.*05*). The t-test results showed that SFA in the autotomy group was significantly higher than that in the control group at 21 d (*P < 0*.*05*) (Table 2).

In the control group, MUFA showed a tendency to rise first and then decline, and it was significantly increased at 14 d compared with 1 d before gradually returning to the initial level; it was significantly lower at 28 d (15.98±1.19%) compared to other times (1 d:24.50±3.30%; 7 d: 21.48±5.46; 14 d: 24.18±2.75; 21 d: 24.83±2.60%) in the autotomy group (*P < 0*.*05*). T-test results showed that MUFA in the autotomy group (28 d: 15.98±1.19%) was significantly lower than that in control group (28 d: 18.95±1.80%) at 28 d (*P < 0*.*05*) (Table 2).

HUFA was significantly decreased at 21 d (15.16±1.41%) compared with 1 d (19.78±1.20%) (*P < 0*.*05*) in the control group, whereas there was no significant change within 28 days in the autotomy group. The T-test results showed that HUFA in the autotomy group was significantly higher than that in the control group (18.81±1.69%) (*P < 0*.*05*) (Table 2). Important long-chain highly unsaturated fatty acids (LCHUFA) also changed during this process, including 22: 6n-3 (DHA), 20: 5n-3 (EPA) and 20: 4n-6 (ARA). Similar to the HUFA trend, DHA decreased significantly at 21 d (*P < 0*.*01*) in the control group and did not significantly change in the autotomy group. The T-test results of DHA showed that DHA in the autotomy group (7 d: 10.71±0.75%) was significantly lower than that in control group (7 d: 12.68±1.39%) at 7 d, whereas it was significantly higher in the autotomy group (: 12.08±1.06%) than in the control group (: 8.75±1.50%) at 21 d (*P < 0*.*05*) (Table 2). Moreover, the T-test results of ARA and EPA showed that there were no significant differences between the autotomy and the control group (Table 2). Finally, the n-3/n-6 in the autotomy group (1.47±0.26%) was significantly increased compared with the control group (1.10±0.11%) at 21 d (*P < 0*.*05*) (Table 2).

#### Muscle moisture and L-tryptophan content

**Fig 8:**
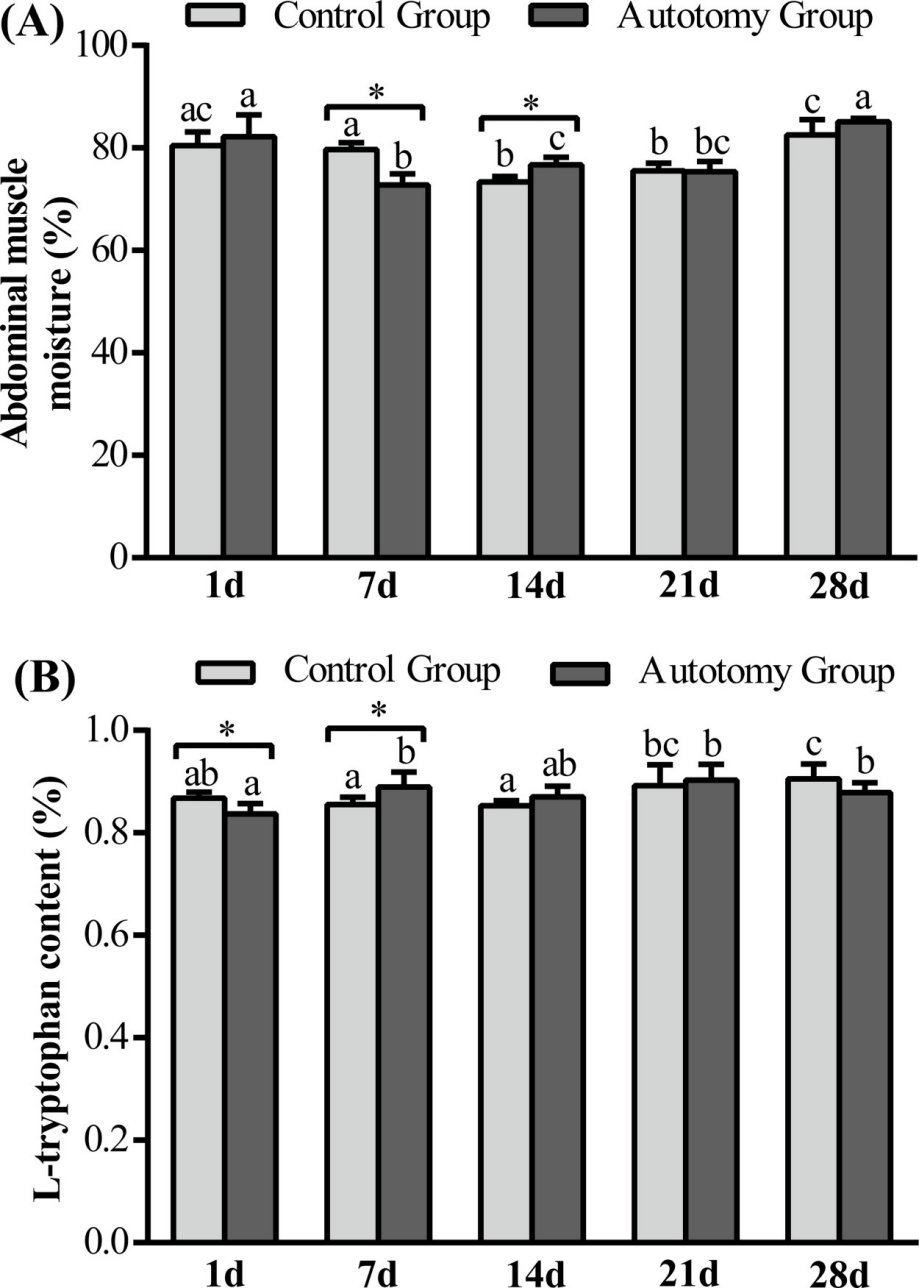
Changes of muscle moisture and L-tryptophan content of *E*. *sinensis* within 28 days after treatment. (A) abdominal muscle moisture; (B) L-tryptophan content. The values are expressed as the means ± SD (n = 5). Different letters above the columns represent significant differences with the same treatment at different times (*P<0*.*05*). * represents significant differences between the control group and the autotomy group at the same time point (* *P < 0*.*05*).

In both the autotomy group and the control group, muscle moisture showed a trend to decrease first and then increase within 28 d (Fig 8A). The muscle moisture decreased significantly at 7 d compared with 1 d and then increased significantly at 14 d and 28 d compared with 7 d in the autotomy group (*P < 0*.*05*). Moreover, compared with the control group, abdominal muscle moisture was significantly lower at 7 d (*P < 0*.*05*) in autotomy group, whereas it was significantly higher at 14 d (*P < 0*.*05*) (Fig 8A).

Muscle L-tryptophan content was significantly increased at 28 d in control group compared with 1 d (*P < 0*.*05*), whereas it was significantly increased at 7 d, and 28 d compared with 1 d after cheliped autotomy (*P < 0*.*05*) (Fig 8B). T-test results showed that the L-tryptophan content was significantly lower in the autotomy group compared with the control group at 1 d (*P < 0*.*05*), whereas it was significantly higher at 7 d (*P < 0*.*05*) (Fig 8B).

## Discussion

### Cheliped bud morphology and composition analysis

Regeneration means the structural and functional reconstruction of an organism for lost tissue or an organ. In the present study, we for the first time observed the morphological changes during the cheliped regeneration of *E*. *sinensis*. Based on previous studies of crustaceans [40,41], we summarized the cheliped regeneration process of *E*. *sinensis* in the following three stages: wound-repair, bud growth and new limb formation. (1) Wound-repair: wound-repair occurs when the cheliped was autotomized, a black substance was spread and deposited in the wound area and gradually formed a layer of dark brown biofilm covering the wound surface to prevent loss of hemolymph and invasion of pathogenic bacteria. We think this black substance may be melanin. Previous studies in crustaceans found that granulocytes were involved in the activation of the prophenoloxidase system (proPO) [42] and then convesion into a PO, thereby gradually inducing the production of melanin, forming an isolation layer by encapsulation and melanization [22], repairing wounds and playing an immune protective role. (2) Bud growth: firstly, transparent hemisphere-like crystalline encrustations broke out of the dark brown biofilm at the autotomy site, and gradually growed into a rod like structure a few days later. The buds were transparent when they first grew out and the surface gradually became black with melanization, but they did not sclerotize. We speculated that the blackening of the bud was due to the melanin sedimentation and melanization.

Studies have reported that active PO participates in the encapsulation and melanization of foreign organisms, as well as in the repair of wounds or sclerotization of new exoskeleton after molting [23,43]. In this experiment, we measured PO activity in HL and CFH during cheliped regeneration process and found that PO activity in the autotomy group was significantly lower than that in the control group. We believe that the decrease of PO activity was related to the large amount of induced melanin production and clearance of wound pathogens. Chitin and crude protein are important components of crab-shell; after the molting of *E*. *sinensis*, proteins related to crab shell formation will be synthesized in large quantities because of the construction of new epidermis [35]. In this study, we found that the crude protein content in cheliped buds increased significantly within 28 days, whereas the chitin content did not change significantly. Moreover, the volume of the cheliped buds continuously increased within 28 days, which contains many protein fibers and resulted in a significant increase in crude protein content. However, the surface of the bud was soft throughout the entire process of cheliped regeneration, and no hardened shell was formed. Therefore, the chitin content did not change significantly during this process. Tian et al. (2013) reported that the chitin content in the shell of *E*. *sinensis* did not change significantly during the molting process [35], which is similar to our result. (3) New limb formation: new limb formation occurred when the cheliped bud continued to grow and the new cheliped was pre-formed in the bud, and thus, the new cheliped was regenerated with the completion of molting. The regenerated cheliped has similar appearance and morphology to the original cheliped, but with a smaller size. With individual growth and after two times of molting, it can generally return to normal size. The cheliped regeneration process of crabs is relatively slow and usually needs to undergo a complete molting cycle in order to grow a complete new limb [12]. The autotomy behavior of *E*. *sinensis* occurs throughout the developmental stage, but regeneration can only occur before sexual maturation. After sexual maturation, the molting will terminate, and they will not be regenerated.

### Growth-related genes

In crustaceans, the ecdysteroid and molt-inhibiting hormone (MIH) jointly regulate the crab’s cheliped regeneration; ecdysteroid is mainly produced by the Y-organs and can promote the cheliped regeneration, and the MIH mainly comes from the eyestalk, which can inhibit the release of ecdysteroid [19]. The process of cheliped regeneration of crabs is relatively slow and usually needs to undergo a complete molting cycle to complete the cheliped regeneration [12]. Therefore, the shortening of the molting cycle means faster completion of limb regeneration. In crustaceans, the molting cycle is regulated by positive regulatory factors (e.g., ecdysteroid, methyl farnesoate) and counter-regulatory factors (e.g., MIH, mandibular organ-inhibiting hormone (MOIH)) [44]. In the present study, we found that most crabs had molting behavior at 21 d, and all crabs in the autotomy group completed molting activity at 28 d. However, the molting rate of control group was only approximately 70%, which indicates that cheliped autotomy can promote molting and shorten the molting cycle of *E*. *sinensis*. Previous studies have shown that limb autotomy can shorten the first molting period of *E*. *sinensis*, *Panulirus longipes* and *Jasus lalandii*, which is consistent with our results. The opposite result was found in studies of *Panulirus argus* and *Scylla serrata* [40,41], which shows that there are differences between different species. In addition, chitinase (Chi) plays an important role in the molting cycle of crabs, which can hydrolyze the chitin from old bones for the synthesis of new bones [21]. Studies have shown that chitinase is involved in many physiological processes in crustaceans, such as morphogenesis, nutrient digestion and pathogen defense [45–47]. Our previous study found that melatonin can promote cheliped regeneration of *E*. *sinensis* by regulating the expression of *EcR*, *MIH* and *Chi* genes [20]. Therefore, in the present study we examined the expression levels of *EcR*, *MIH* and *Chi* genes during the cheliped regeneration process of *E*. *sinensis*. The results showed that the expression of *EcR*-mRNA in the tissues was significantly higher in the autotomy group than that in the control group, whereas the expression of *MIH*-mRNA was significantly lower in the autotomy group. The up-regulation of *EcR*-mRNA and down-regulation of *MIH*-mRNA promoted the molting and cheliped regeneration of *E*. *sinensis*. Moreover, we observed a similar trend in the expression of *Chi*-mRNA and *EcR*-mRNA in tissues, which is consistent with the study of *Fenneropenaeus chinensis* [48]. During the process of cheliped regeneration, the expression levels of Chi-mRNA were significantly increased in tissues, which showed that Chi played an important role in cheliped regeneration and the hardening of new shells after molting. A previous study reported that the expression of Chi-mRNA is significantly up-regulated before molting, which was significantly higher than during other molting periods[49], consistent with our findings.

### Nutritional status

#### Hepatopancreas total lipid and fatty acid composition

He *et al*. (2013, 2016) believed that the main purpose of storage nutrition in crustaceans is for molting and growth, and to speed up the regeneration of new limbs, the juvenile crabs may begin to molt only with part of nutrients stored, thus resulting in a shortened molting cycle [12,50]. For *E*. *sinensis*, lipids are the most important energy reserve and biofilm structural material in the body [26]. Hepatopancreas, as the main lipid storage organ and metabolic center, is one of the main energy sources for the molting cycle and has an important connection with the formation of new exoskeleton during molting cycle and metabolism during the soft-shell stage after molting and when crustaceans stop feeding [37,51]. In the present study, we observed that the hepatopancreas total lipid content was significantly decreased in both the autotomy group and control group, and it was significantly lower in the autotomy group than control group at 14 d and. To regenerate new cheliped as soon as possible, crabs in the autotomy group began molting earlier in comparison with the control group. Most of the crabs in the autotomy group had achieved molting at; cheliped regeneration and reconstitution of new epidermis after molting required a large amount of energy, which resulted in a decrease of the total lipid content, consistent with the results of Ma *et al*. (2014) [24]. SFA and MUFA are important fatty acid components, the main function of which is to provide energy [26]. In this study, compared with the control group, MUFA was significantly decreased in the autotomy group at 28 d, indicating that MUFA is the main energy-supplying material in the process of cheliped regeneration. Ma *et al*. (2014) believed that the energy provided by SFA and MUFA was not proportional in the process of molting of *E*. *sinensis*; MUFA was significantly more consumed [24], which is similar to our results. PUFA and HUFA are present as the main components of phospholipids in biofilms rather than as energy-supplying material [35]. In the present study, ∑ n-6 PUFA and ∑ n-3 PUFA were significantly lower in the autotomy group than in control group at 1 d and 7 d; we speculated that it was transferred to construct new biofilms for regenerating buds. Highly unsaturated fatty acids (HUFA) such as ARA, EPA and DHA are the main components of phospholipids in the membrane structure [26], which plays an important role in the metabolism of *E*. *sinensis*. After DHA, EPA and ARA are used, they must be supplemented in time to maintain normal metabolic processes of the organism. In our study, although there was no significant difference of ARA and EPA between the autotomy group and the control group, the DHA level was significantly lower at 7 d and significantly higher at in the autotomy group than in the control group. The DHA decreased significantly at 7 d in the autotomy group because the growth of the regenerated cheliped consumed a large amount of DHA at 7 d, whereas the DHA level was significantly increased at due to the molting of crabs in the autotomy group; in order to maintain the normal life activities of crabs, DHA was greatly supplemented. HUFA also showed similar results. Our study found that the molting of crabs in the autotomy group was earlier than in control group; in order to promote cheliped regeneration and molting, it is possible to enhance the hepatopancreas total lipid content and to strengthen the nutrition of fatty acids such as MUFA and DHA, which will help to promote the cheliped regeneration and improve survival rate after molting.

#### Muscle L-tryptophan content and muscle moisture

In addition, L-tryptophan, as an essential amino acid for crustaceans [27], participates in protein and lipid metabolism of animals [28]. Studies have reported that L-tryptophan can improve animal growth performance, feed conversion efficiency and intestinal digestive capacity [52]. Moreover, tryptophan is the precursor of melatonin, which can be converted into melatonin in vivo [33,34], and plays an important role in the antioxidant function and immune regulation of the organism [29,30]. In the present study, the abdominal muscle L-tryptophan content was significantly lower at 1 d and significantly higher at 7 d in the autotomy group compared with the control group, which indicates that L-tryptophan participates in immunoregulation at 1 d to cope with the cheliped autotomy stress. Our previous study found that the immunity of *E*. *sinensis* was significantly reduced after cheliped autotomy in the short term [4]. Studies have reported that tryptophan and melatonin can enhance the anti-stress ability of *Apostichopus japonicus* Selenkaand and *Solea senegalensis* (Senegalese sole) [53,54]. However, the present study found that L-tryptophan content increased significantly in the autotomy group at 7 d; we speculate that the main task of L- tryptophan at this time is not anti-stress and immunity regulation but instead to promote cheliped regeneration, so the body began to absorb and convert to L-tryptophan. Our previous study found that melatonin injection in *E*. *sinensis* can up-regulate the expression of *EcR*-mRNA and *Chi*-mRNA and down-regulate the expression of *MIH*-mRNA in tissues to promote cheliped regeneration [20]. L-tryptophan, as a precursor of melatonin, accelerates the conversion of substances and indirectly promotes the expression of growth-related genes and accelerates cheliped regeneration of *E*. *sinensis*. In addition, our study found that in the autotomy group, compared with 1 d, muscle moisture decreased significantly at 7 d and then increased significantly at 14 d, which is contrary to the changing trend of muscle L-tryptophan content and the main energetic substance MUFA in the hepatopancreas. A previous study reported that in the absence of nutrition, crustaceans could use water to fill in the energy-consuming substances in tissues [32]. He *et al*. (2013) found similar results in the study of *E*. *sinensis* and speculated that it might be a common feature of the physiological regulation of crustaceans [50].

## Conclusion

In the present study, we found that the new cheliped was pre-formed in the bud and then regenerated with the completion of the molting of *E*. *sinensis*. During the cheliped regeneration process, crabs could accelerate bud growth by increasing the crude protein content, promote regeneration rate and shorten molting cycle by up-regulating the positive growth-related gene and down-regulating the molting inhibition gene, and accelerate nutrient metabolism such as lipid metabolism and tryptophan metabolism. Our study found that the molting of crabs in the autotomy group was earlier than in control group. In order to promote the cheliped regeneration and molting, we recommend that we should enhance the hepatopancreas total lipid content, strengthen the nutrition of fatty acids such as MUFA and DHA and supply appropriate amounts of L-tryptophan to the diet, which will help to promote cheliped regeneration and improve survival rate after molting.

## Supporting information

S1 Dataset Data. (ZIP)
