# Supplementary figures and images for: Changes in bud morphology, growth-related genes and nutritional status during cheliped regeneration in the Chinese mitten crab, Eriocheir sinensis
Source: PLoS One. 2018 Dec 26;13(12):e0209617. doi: 10.1371/journal.pone.0209617 (PMC6306232; doi:10.1371/journal.pone.0209617)

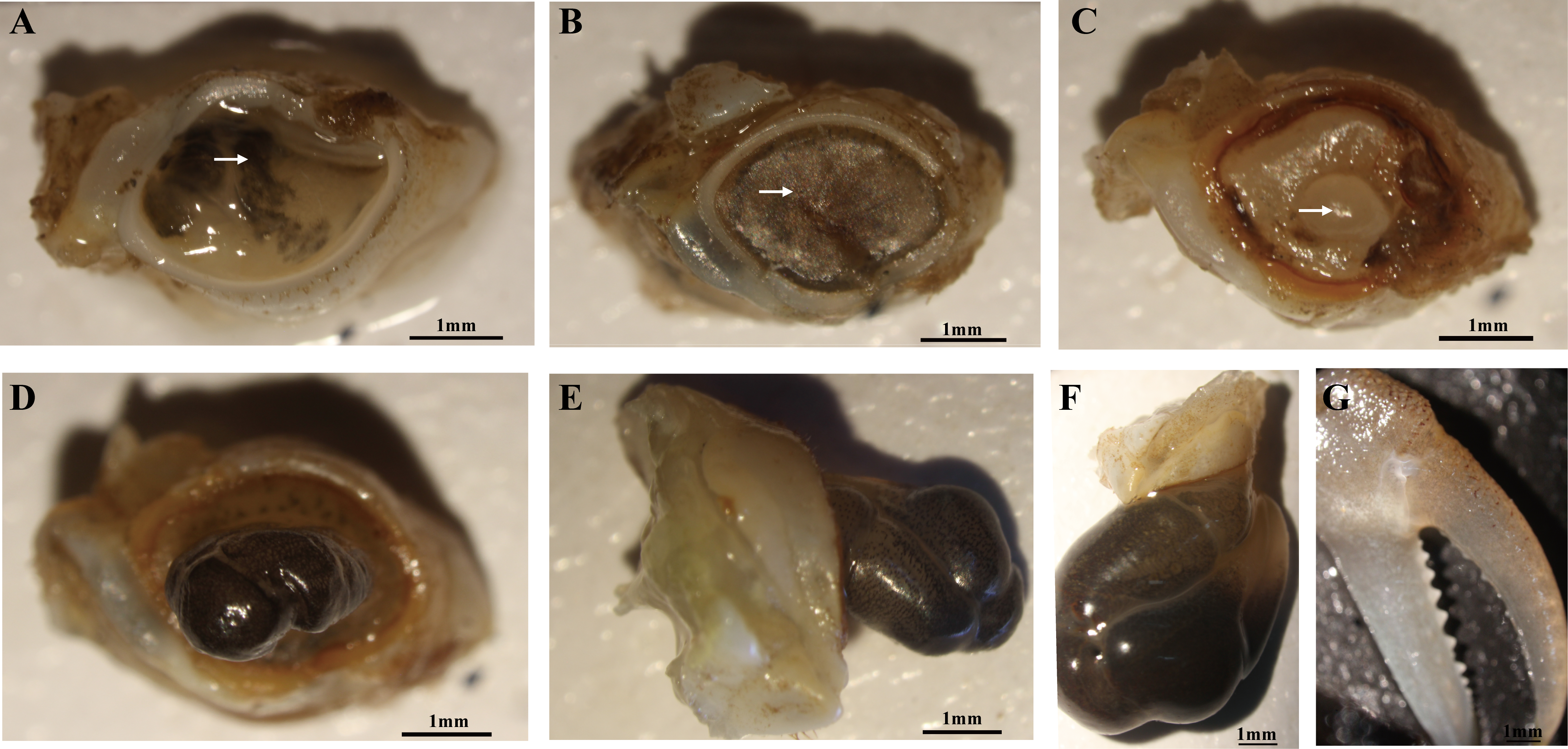

Supplement: S1 Dataset — (ZIP) [file pone.0209617.s001.zip › data/Fig 1/cheliped bud.tif]
